# High Mobility Group Box-1 Promotes Inflammation-Induced Lymphangiogenesis via Toll-Like Receptor 4-Dependent Signalling Pathway

**DOI:** 10.1371/journal.pone.0154187

**Published:** 2016-04-21

**Authors:** Longhui Han, Minglian Zhang, Mengmeng Wang, Jinchen Jia, Miying Zhao, Yiming Fan, Xiaorong Li

**Affiliations:** 1 Tianjin Medical University Eye Hospital/Eye Institute, School of Optometry and Ophthalmology, Tianjin Medical University, Tianjin, China; 2 Hebei Provincial Key Laboratory of Ophthalmology, Hebei Provincial Eye institute, Hebei Provincial Eye Hospital, Xingtai, Hebei, China; Ottawa Hospital Research Institute, CANADA

## Abstract

Lymphangiogenesis in inflammation has received considerable attention in recent years. Administration of modulating lymphangiogenesis provides more possibilities of treating inflammation-associated diseases. However, the main mediators and factors governing inflammation-induced lymphangiogenesis (ILA) are yet to be defined. Here, we explored the role of HMGB1-TLR4 signalling pathway in modulating inflammation-induced lymphangiogenesis and its underlying mechanisms using an ILA mouse model and 2 cell lines. Our results show that HMGB1 promoted VEGF-C-induced HDLECs proliferation in a dose-dependent manner and TLR4 mediates HMGB1-induced LECs proliferation and tube formation *in vitro*. And *in vivo*, rHMGB1 treatment significantly promoted ILA, and the promoting effects was inhibited notably when HMGB1-TLR4 was blocked. HMGB1-associated ILA is primarily dependent on TLR4 but not on TLR2. In mechanisms, the recruitment and activation of CD11b+ cells are important cellular mechanisms in HMGB1-TLR4 associated ILA, and multiple key pro-lymphangiogenesis molecules mediates HMGB1-TLR4 associated ILA, including VEGF-C/VEGFR3, inflammatory factors IL-1β and TNF-α, MMP-2 and MMP-9 and NF-κB p65. In conclusion, HMGB1-associated ILA is primarily dependent on TLR4, and CD11b+ cells and multiple molecular mechanisms mediate HMGB1-TLR4 associated ILA. Furthermore, the ILA can be effectively modulated by HMGB1-TLR4 signalling. Consequently, administration of modulating ILA through HMGB1-TLR4 pathway may provide us more possibilities of treating inflammation and lymphangiogenesis associated diseases.

## Introduction

Lymphangiogenesis (the growth of new initial lymphatics) in inflammation has received considerable attention in recent years. Lymphangiogenesis may be triggered by inflammation during various inflammation-associated pathological processes [1]. More importantly, lymphangiogenesis may affect the process of inflammation. Therefore, administration of modulating lymphangiogenesis provides more possibilities of treating inflammation-associated diseases [2]. But, the main mediators and factors governing inflammation-induced lymphangiogenesis (ILA) are yet to be defned [3].

High mobility group box-1 (HMGB1) protein was originally identified as a nuclear factor. In recent years, HMGB1 is also recognized as Inflammatory factors, which can be actively secreted from immune cells or cancer cells, or can be passively released from necrotic or damaged cells [4–8]. Accumulating evidence suggests that HMGB1 strongly correlates with inflammation and lymphangiogenesis [4, 9–16]. In vitro, HMGB1 induced the proliferation, migration, and tube formation of lymphatic endothelial cells (LECs) [13]. In vivo, HMGB1 promoted lymphangiogenesis in the pathological process of cancer [14–16]. HMGB1 blockade significantly reduced inflammatory lymphangiogenesis in inflamed draining lymph nodes [17].

Toll-like receptor 4 (TLR4) is one of the key pattern recognition receptors (PRRs) that recognize pathogen-associated molecular patterns (PAMPs) and damage-associated molecular patterns (DAMPs) to initiate inflammation and innate immune. Recent researches showed that TLR4 plays an important role in lymphangiogenesis. TLR4 in lymphatic endothelial cells (LECs) plays an essential role in LPS-induced inflammatory lymphangiogenesis by enhancing the recruitment and infiltration of macrophages [18]. TLR4-expressing tumors may activate systemic inflammatory circuits that promote lymphangiogenesis and metastasis [19]. TLR4 deficiency is associated with decreased lymphangiogenesis and reduced macrophage infiltration after lymphatic injury [20].

TLR4 is one of the main receptors of HMGB1 [21–25]. HMGB1-TLR4 pathway contributes to inflammation by multiple mechanisms [26, 27]. At the same time, HMGB1 and TLR4 strongly correlates with inflammation and lymphangiogenesis as described above. Therefore, we postulate that HMGB1-TLR4 may be a crucial signaling pathway in modulating inflammation-induced lymphangiogenesis. To test this hypothesis, we used a mouse model of ILA and 2 cell lines, which shows that HMGB1 promotes inflammation-induced lymphangiogenesis via TLR4-dependent signalling pathway, and HMGB1-TLR4 promotes inflammation-induced lymphangiogenesis by recruiting and activating of CD11b^+^ cells and up-regulating of pro-lymphangiogenesis molecules. Furthermore, inflammation-induced lymphangiogenesis was inhibited when HMGB1-TLR4 pathway was blocked.

Our results indicate for the first time that HMGB1-associated ILA is primarily dependent on TLR4, and CD11b^+^ cells and multiple molecular mechanisms mediate HMGB1-TLR4 associated ILA. More importantly, the ILA can be effectively modulated by HMGB1-TLR4 signalling. This may provide us more possibilities of treating inflammation and lymphangiogenesis associated diseases.

## Materials and Methods

### Ethics statement and animals

This study strictly adhered to the Association for Research in Vision and Ophthalmology’s Statement for the Use of Animals in Ophthalmic and Vision Research and was approved and monitored by the Institutional Animal Care and Use Committee of Hebei Provincial Eye Hospital. Adult male C57BL/10 mice, C57BL/10-based TLR2^-/-^ mice and C57BL/10-based TLR4^-/-^ mice were obtained from the Model Animal Research Center of Nanjing University, maintained under a 12-h light/dark cycle in a temperature- and humidity-controlled room and given ad libitum access to food and water. Male mice aged 8 to 10 weeks (21–25 g) were used for experiments. Surgeries were performed under chloral hydrate solution anesthesia. All efforts were taken to minimize animals’ suffering.

### Antibodies and reagents

The antibodies, including anti-lymphatic vessel endothelial hyaluronan receptor-1 (LYVE-1) polyclonal, anti-VEGF-C monoclonal, anti-VEGF receptor 3 (VEGFR3) monoclonal, anti-CD11b monoclonal, anti-F4/80 monoclonal, and Anti- proliferating cell nuclear antigen (PCNA) monoclonal, were from abcam (Hong Kong, China). And the other antibodies, including anti-nuclear factor-kappa B (NF-κB) p65 monoclonal, anti-phosphorylated NF-κB p65 monoclonal, anti-β-actin monoclonal, horseradish peroxidase (HRP)-conjugated anti-mouse secondary antibody, HRP-conjugated anti-rabbit secondary antibody, Alexa Fluor 488-coupled goat anti-rat secondary antibody, Alexa Fluor 555-coupled goat anti-rabbit secondary antibody, were from Cell Signaling Technology Inc. (Danvers, MA). Recombinant HMGB1 (rHMGB1) and Box-A (a specific antagonist of HMGB1) were purchased from IBL International (Hamburg, Germany). VEGF-C was from Sigma (St. Louis, MO, USA).

### ILA model and treatment

A mouse model of ILA in the cornea was used as previously described [28, 29]. In brief, each mouse was deeply anesthetised with intraperitoneal injection of 10% chloral hydrate solution (0.04mL/10g) prior to surgery. A circle was marked with a 2-mm corneal trephine on the centre of the cornea in the right eye. Then three “8”-shaped intrastromal sutures were made using 11–0 nylon sutures (Jiahe Inc., Taibei, Taiwan, China; NT0411), and each suture extended to approximately 120° of the corneal circumference. Then the ILA model mice were randomly divided into each group (n = 5 per group) and were given topical administration of 5 μL rHMGB1 (80μg/mL), A-box (100 μg/mL) or vehicle (sterile saline solution) to the right eye twice a day. The *in vivo* experiments lasted for 10 days and were repeated at least 3 times independently.

### Immunostaining and quantification

The whole-mount corneal LYVE-1 immunostaining was performed as previously described [28, 30]. In brief, the mice were sacrificed at the end of the 10-day experiment. The eyes were fixed with 4% (wt/vol) paraformaldehyde at 4°C overnight. The corneas were excised under a biomicroscope and blocked with 3% bovine serum albumin (BSA) in PBST (0.3% (vol/vol) Triton X-100 in PBS) for 2 hours, followed by incubating overnight at 4°C with anti-LYVE-1 (1:200). On the second day, the tissue was incubated with Alexa Fluor 555-coupled goat anti-rabbit antibody (1:400) for 2 hours at room temperature. Cornea flat mounted on microscope slides with Vectashield mounting medium was then examined with a fluorescent microscope, and images were taken with a 5× objective. Finally, the lymphatic vessels were quantified in Adobe Photoshop CC programme as previously described [28].

The frozen-section corneal immunostaining was made as previously described [28]. In brief, the mice were sacrificed at the end of the 10-day experiment. The eyeballs were cut into 6-micron-thick frozen sections, fixed with 4% (wt/vol) paraformaldehyde for 10 min and incubated with anti-CD11b antibody (1:400), followed by the secondary antibody Alexa Fluor 488-coupled goat anti-rat (1:400).

### Cell culture

Human dermal lymphatic endothelial cells (HDLECs) were purchased from ScienCell (Carlsbad, CA) and maintained in endothelial cell basal medium-2 with growth supplements (EBM-2 MV). For the proliferation assay, HDLECs were pre-incubated with different doses of HMGB1 (0 ng/mL, 50 ng/mL, 100 ng/mL, 500 ng/mL, 1000 ng/mL or 2000 ng/mL) for 1 hour, followed by incubation with or without VEGF-C (10 ng/mL). The cells were allowed to proliferate for 24 hours, and 100 μL of cells from each well was transferred to a new 96-well plate with 10 μL of Cell Counting Kit-8 solution (Dojindo Laboratories, Kumamoto, Japan). The absorbance at 450 nm was then measured using a microplate reader.

The RAW264.7 murine macrophage cell line were purchased from Shanghai Cell Bank of Academia Sinica (Shanghai, China) and cultured in Dulbecco’s modified Eagle’s medium supplemented with 2 mM L-glutamine and 10% heat-inactivated foetal bovine serum (FBS).

### Knock-down of TLR4 by siRNA transfection

HDLECs or RAW264.7 cells in a 6-well plate (approximately 50% confluence) were transfected with 180 pmol of TLR4-specific siRNA (Santa Cruz Biotechnology, Inc. Santa Cruz, USA). After 48 hours of transfection, the cells were used for further experiments.

### ELISA

The concentrations of TNF-α and IL-1β in the culture medium of RAW264.7 cells were detected using ELISA kits (eBioscience, San Diego, CA). Briefly, an ELISA plate was coated with 100 μL/well of capture antibody and incubated overnight at 4°C. Then, 100 μL/well of supernatant of cultured cells was added to the appropriate wells and incubated at room temperature for 2 hours. Finally, Read plate at 450 nm and analyse data.

### Real-Time PCR

The corneas or RAW264.7 cells from each experimental group were used for the real-time PCR analysis. Total RNA from the tissue or cell lysates were extracted with an RNeasy Mini Kit (Qiagen, Valencia, CA), and cDNA was generated using an Omniscript RT Kit (Qiagen). The mRNA expression was then quantified using ABsolute SYBR Green ROX mix (Thermo, Waltham, MA). All samples were run in at least triplicate, and the relative expression of IL-1β, TNF-α or VEGF-C was determined by normalising the expression of each target to the internal reference β-actin using the 2^-ΔΔCT^ method. The primer (Invitrogen, Shanghai, China) sequences, hybridation temperatures, number of cycles, and the length of the Real-time PCR products are listed in S1 Table.

### Nuclear protein extraction

The extraction and isolation of nuclear protein was performed according to the Nuclear and Cytoplasmic Protein Extraction Kit (Beyotime, Jiangsu, China) and Wang’s protocol [31].

### Western blot analysis

Lysates from 2 pooled mouse corneas or RAW264.7 cells (50 μg of total protein or nuclear protein) were separated on a polyacrylamide-SDS gel and electro-blotted onto a nitrocellulose membrane (Bio-Rad, Hercules, CA, USA). The membrane was blocked with 5% nonfat milk, and then was incubated with antibodies against VEGF-C, VEGFR3, NF-κBp65, p-NF-κBp65 and β-actin (for total protein)/PCNA (for nuclear protein) (1:1000), followed by incubation with an HRP-conjugated secondary antibody (1:3000). The signals were visualised using enhanced chemiluminescence detection (Pierce, Rockford, IL). All experiments were performed in at least triplicate. Quantification of Western blots was performed using Adobe Photoshop CC programme.

### MMPs activity assays

MMP-2 and MMP-9 activity in corneas or RAW264.7 cells was measured using a fluorogenic peptide substrate (R&D Systems) according to the manufacturer’s protocol. In brief, the MMPs substrate was diluted in TCN buffer (50 mmol/L Tris-HCl, 10 mmol/L CaCl_2_ and 150 mmol/L NaCl; pH 7.5) and added to supernatants from corneal or RAW264.7 cell lysates (pre-activated by aminophenylmercuric acetate for 1 hour). 30 minutes later, total MMP activity was determined with FLX 800 Microplate Fluorescence Reader (Bio-Tek Instruments, Winooski, VT).

### Statistical analysis

All statistical analyses were performed using SPSS 21.0 software (SPSS Inc., Chicago, IL). Normal distribution of the data was tested with Kolmogorov-Smirnov tests. All data in figures are presented as mean ± SD. Student’s t-test was performed for analysing differences between two groups. Differences were considered statistically significant when a *p* value was < 0.05.

## Results

### The expression of HMGB1 and TLR4 increases in the ILA model

**Fig 1:**
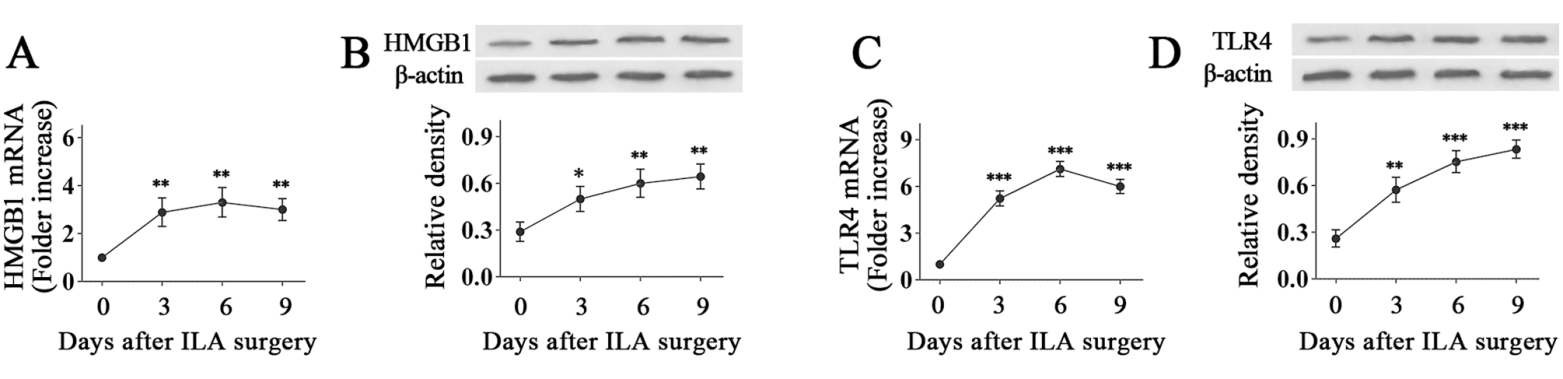
The expression of HMGB1 and TLR4 increases in the ILA model. (A and B): Real-time PCR and Western blot show the increased expression of HMGB1 in the inflamed corneas after the ILA surgery. (C and D): Real-time PCR and Western blot show the increased expression of TLR4 in the inflamed corneas after the ILA surgery was performed. *p < 0.05, **p < 0.01, ***p < 0.001 (compared with normal corneas).

To explore the relationship between HMGB1, TLR4 and ILA, we first determined the expression of HMGB1 and TLR4 in the inflamed cornea after ILA surgery was performed. Real-time PCR and Western blot show that the expressions of HMGB1 and TLR4 increased in the ILA corneas compared with the normal corneas at both mRNA and protein levels, which was sustained over 9 days (Fig 1A–1D).

### TLR4 mediates HMGB1-induced LECs proliferation and tube formation

**Fig 2:**
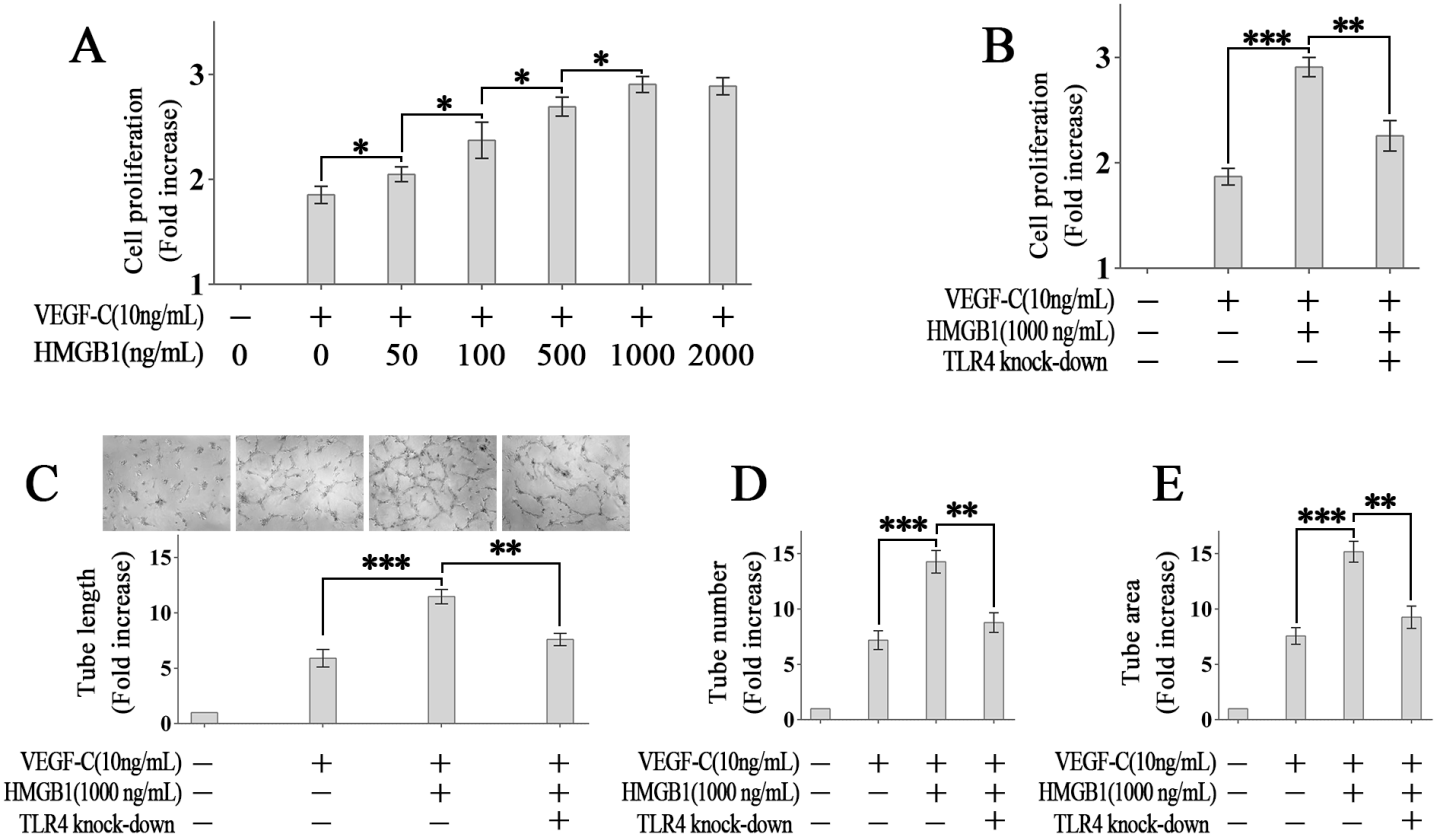
TLR4 mediates HMGB1-induced LECs proliferation and tube formation. (A): HMGB1 promoted VEGF-C-induced HDLECs proliferation in a dose-dependent manner. (B): TLR4 mediates HMGB1-induced LECs proliferation. (C-E): TLR4 mediates HMGB1-induced LECs tube formation.*p < 0.05, **p < 0.01, ***p < 0.001

In vitro, we tested whether HMGB1 could promote the proliferation of lymphatic endothelial cells (LECs). Our results show that HMGB1 promoted VEGF-C-induced HDLECs proliferation in a dose-dependent manner (Fig 2A). In particular, VEGF-C-stimulated HDLECs proliferation was promoted by 1000 ng/mL rHMGB1 to approximate the highest level. No more promoting effect was observed by doubling the concentration of rHMGB1 to 2000 ng/mL. Knock-down of TLR4 by TLR4-specific siRNA inhibited the promoting effect of HMGB1 significantly (Fig 2B), indicating that TLR4 mediates HMGB1-induced LECs proliferation.

Then we tested the role of HMGB1 and TLR4 in LECs tube formation. Our results show that the tube length, tube number and tube area were all promoted by rHMGB1 stimulation, and the promoting effects were inhibited by TLR4 knock-down (Fig 2C–2E).

### TLR4 mediates HMGB1-associated ILA

**Fig 3:**
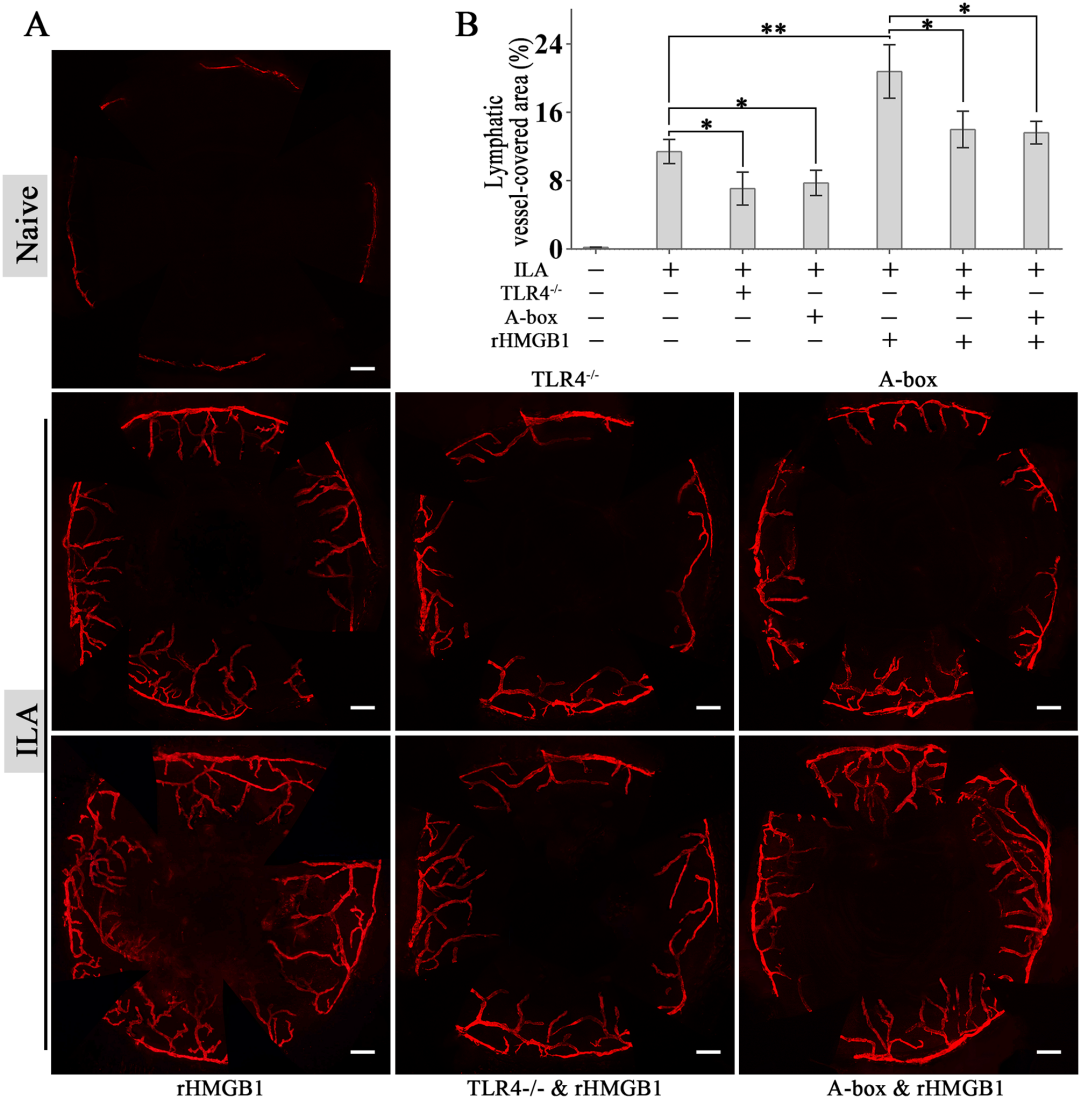
TLR4 mediates HMGB1-associated ILA. (A and B): Representative images and quantification of LYVE-1-labelled corneal lymphangiogenesis in different groups, which shows that TLR4 mediates HMGB1-associated ILA. *p < 0.05, **p < 0.01. The scale bars represent 300 μm.

In vivo, rHMGB1 treatment significantly promoted the ILA in mouse corneas (Fig 3A and 3B). Knock-out of TLR4 or antagonizing HMGB1 by specific antagonist A-box inhibited ILA notably. Furthermore, the inhibiting effects were still effective when the mice were treated with rHMGB1 at the same time (Fig 3A and 3B).

### HMGB1-associated ILA is not dependent on TLR2

**Fig 4:**
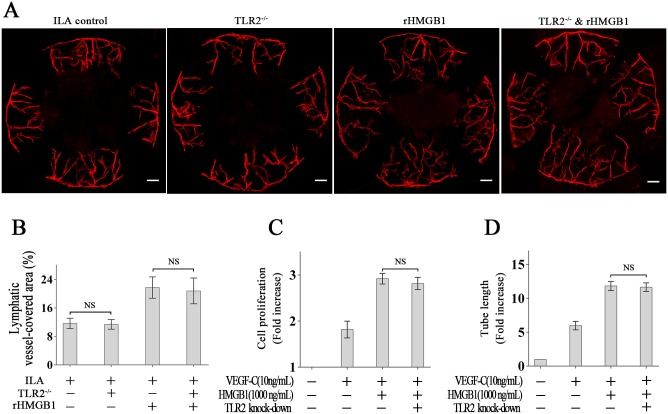
HMGB1-associated ILA is not dependent on TLR2. (A and B): Representative images and quantification of LYVE-1-labelled corneal lymphangiogenesis in different groups, which shows that TLR2 knockout is not significant in HMGB1-induced ILA. (C): TLR2 is not significant in HMGB1-induced LECs proliferation. (D): TLR2 is not significant in HMGB1-induced LECs tube formation. The scale bars represent 300 μm.

TLR2 has also been reported to recognize HMGB1 [32], so we analyzed its role in HMGB1-associated ILA using TLR2^-/-^ mice. We found that ILA developed in TLR2^-/-^ mice was not different from that in WT mice, regardless of the application of rHMGB1 or not (Fig 4A and 4B). In vitro, TLR2 knockout is not significant in HMGB1-induced LECs proliferation and tube formation (Fig 4C and 4D).

These data suggest that HMGB1-associated ILA is primarily dependent on TLR4 but not on TLR2.

### The recruitment and activation of CD11b^+^ cells are important cellular mechanisms in HMGB1-TLR4 associated ILA

**Fig 5:**
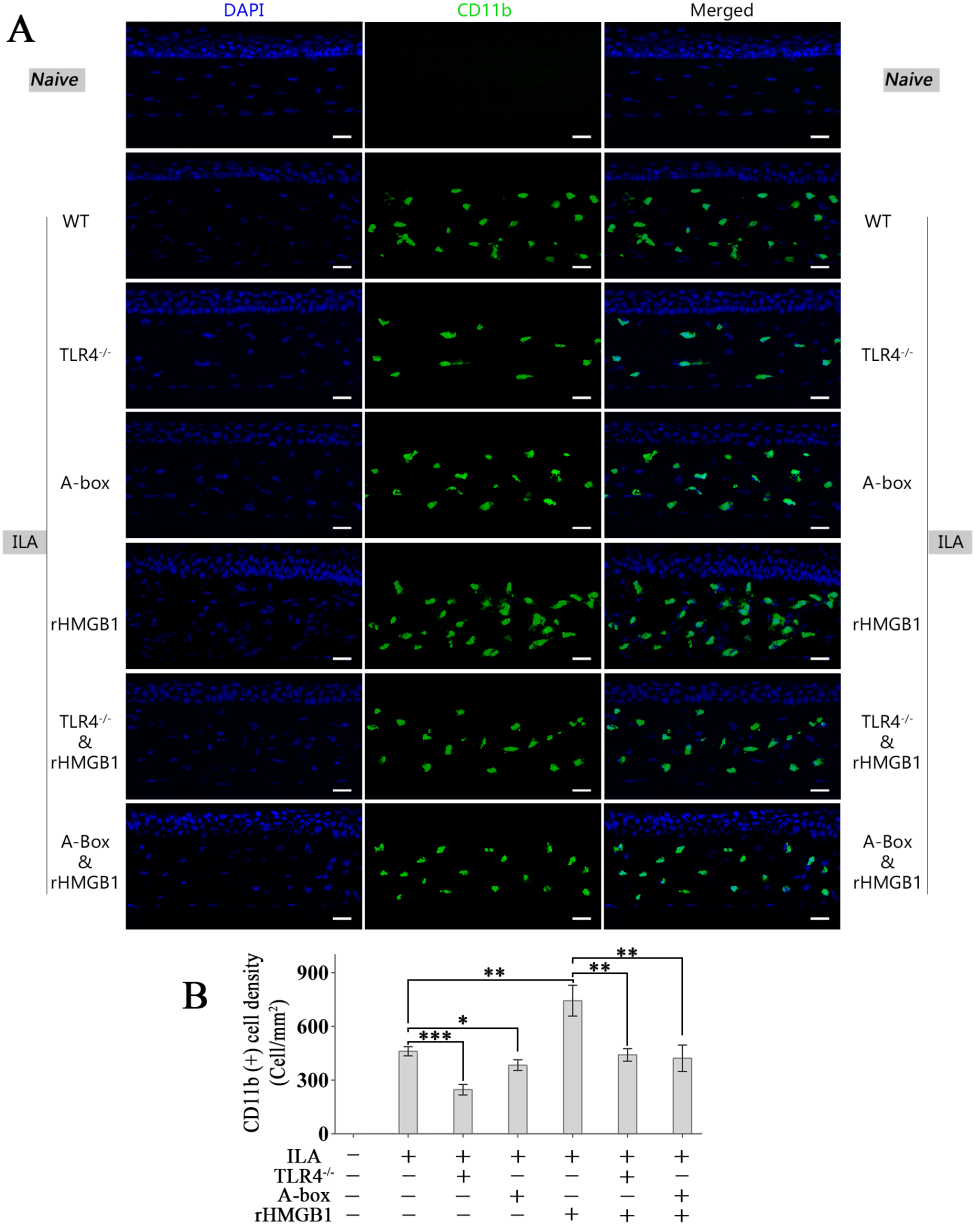
The recruitment of CD11b^+^ cells is important cellular mechanisms in HMGB1-TLR4 associated ILA. (A): Representative images of CD11b^+^ cells in corneas of different groups. (B): Quantification of CD11b^+^ cells in corneas. *p < 0.05, **p < 0.01, ***p < 0.001. The scale bars represent 25 μm.

The number of CD11b^+^ cells in ILA corneas with rHMGB1 treatment significantly increased compared with the number in no rHMGB1-treatment ILA corneas (Fig 5A and 5B). Knock-out of TLR4 or antagonizing HMGB1 by A-box inhibited the recruitment of CD11b^+^ cells notably with or without the rHMGB1-stimulation (Fig 5A and 5B).

**Fig 6:**
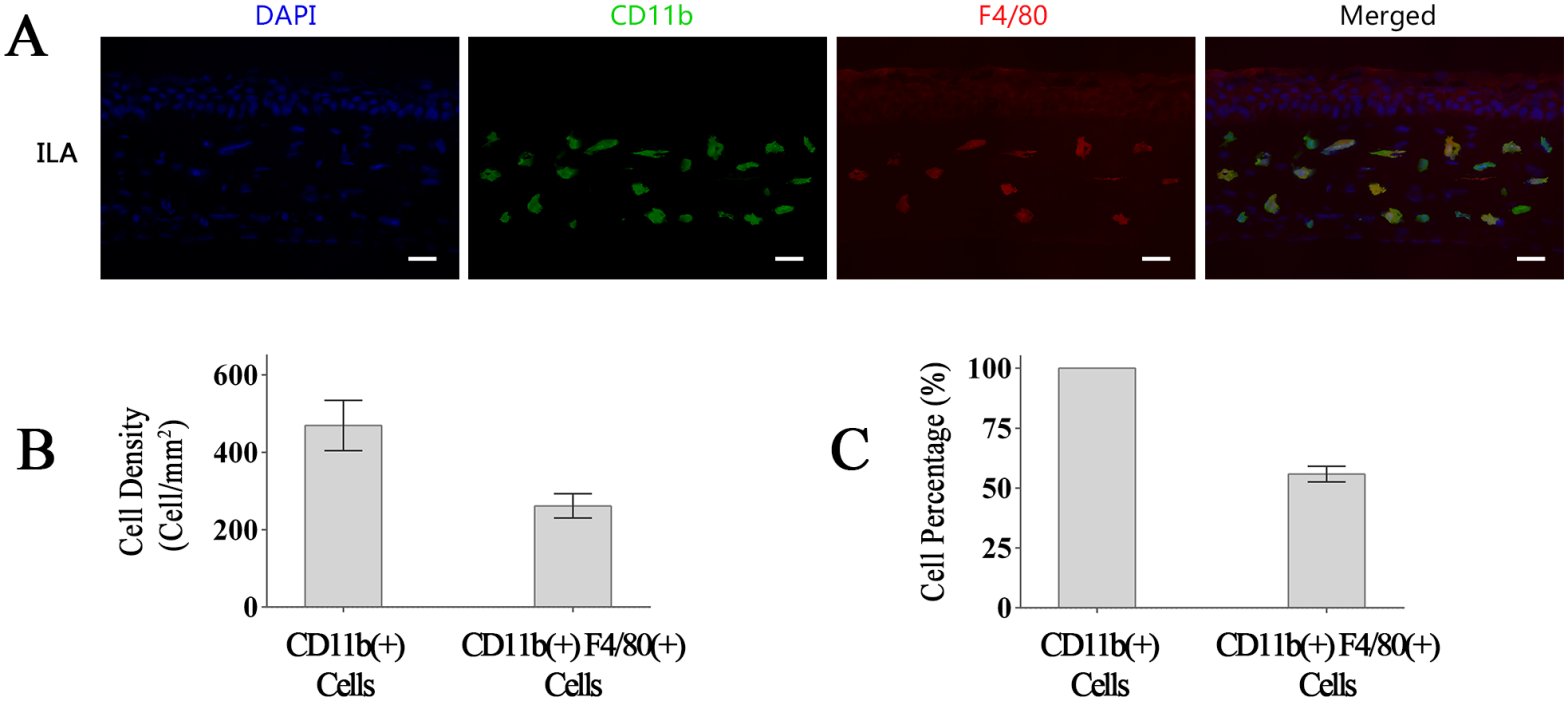
Macrophages are major CD11b^+^ cells in the ILA model. (A): Representative images of CD11b^+^ F4/80^+^ cells in the corneas of ILA mice. (B): Quantification of CD11b^+^ cells and CD11b^+^ F4/80^+^ cells in the ILA corneas. (C): macrophages (CD11b^+^ F4/80^+^ cells) are major CD11b^+^ cells (over half) in the ILA model. The scale bars represent 25 μm.

CD11b is expressed in several types of leukocytes, mainly including macrophages/monocytes and granulocytes [33–35]. Among them, macrophages are essential for pathological lymphangiogenesis [36]. Therefore, we tested whether Macrophages are major CD11b^+^ cells in the ILA model. Our results demonstrate that over half of the CD11b^+^ cells in the ILA corneas are macrophages (CD11b^+^ F4/80^+^ cells) (Fig 6A–6C).

Then we explored the role of HMGB1-TLR4 in the activation of macrophages in vitro. We tested some pro-lymphangiogenesis molecules using the RAW264.7 cells.

**Fig 7:**
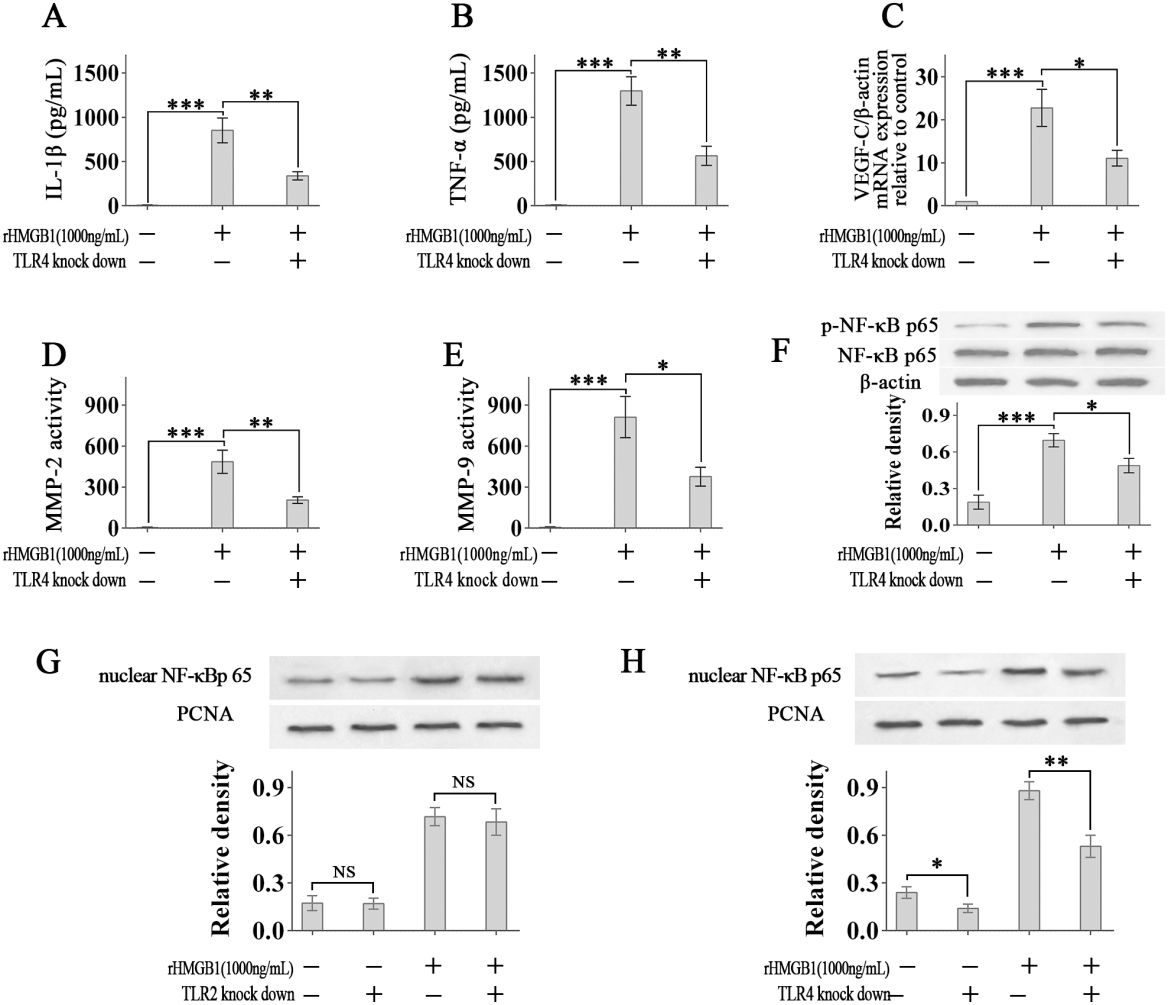
HMGB1-TLR4 signalling pathway plays an important role in the activation of macrophages. (A and B): The levels of IL-1β and TNF-α in the supernatants of RAW264.7 cells were measured using ELISA. (C): The expression of VEGF-C mRNA was determined using real-time PCR. (D and E): MMP-2 and MMP-9 activity in RAW264.7 cells lysates. (F): The levels of phosphorylated and total NF-κB p65 were determined using Western blotting. (G and H): The levels of nuclear NF-κB p65 were determined using Western blotting. *p < 0.05, **p < 0.01, ***p < 0.001.

ELISA analysis shows that rHMBG1-treatment significantly increased IL-1β and TNF-α release by RAW264.7 cells (Fig 7A and 7B). And knock-down of TLR4 by siRNA significantly inhibited the IL-1β and TNF-α release (Fig 7A and 7B). Additionally, real-time PCR shows that rHMBG1-treatment significantly increased the expression of VEGF-C mRNA, which was inhibited by TLR4 knock-down (Fig 7C). Next, MMP-2 and MMP-9 activity was analyzed. Our results show that rHMBG1-treatment significantly increased MMP-2 and MMP-9 activity, which was also inhibited by TLR4 knock-down (Fig 7D and 7E).

Further, the levels of phosphorylated and total NF-κB p65 were detected by western blotting. The results show that rHMBG1-treatment significantly increased the phosphorylation of NF-κB p65 in cells and knock-down of TLR4 significantly inhibited the phosphorylation (Fig 7F).

The effect of HMGB1 in nuclear translocation of NF-κB p65 was also detected. Western blot results show that HMGB1 increased the nuclear NF-κB p65 significantly (Fig 7G and 7H), and knockdown of TLR4 inhibited the nuclear translocation of NF-κB p65 in the presence or absence of HMGB1, but the inhibiting effect was more significant in the presence of HMGB1 (Fig 7H). TLR2 knock down did not affect the nuclear translocation of NF-κB p65 in the presence or absence of HMGB1 (Fig 7G).

### Multiple molecular mechanisms mediates HMGB1-TLR4 associated ILA

To explore the molecular mechanisms of HMGB1-TLR4 in modulating ILA, we tested several main factors involved in lymphangiogenesis.

**Fig 8:**
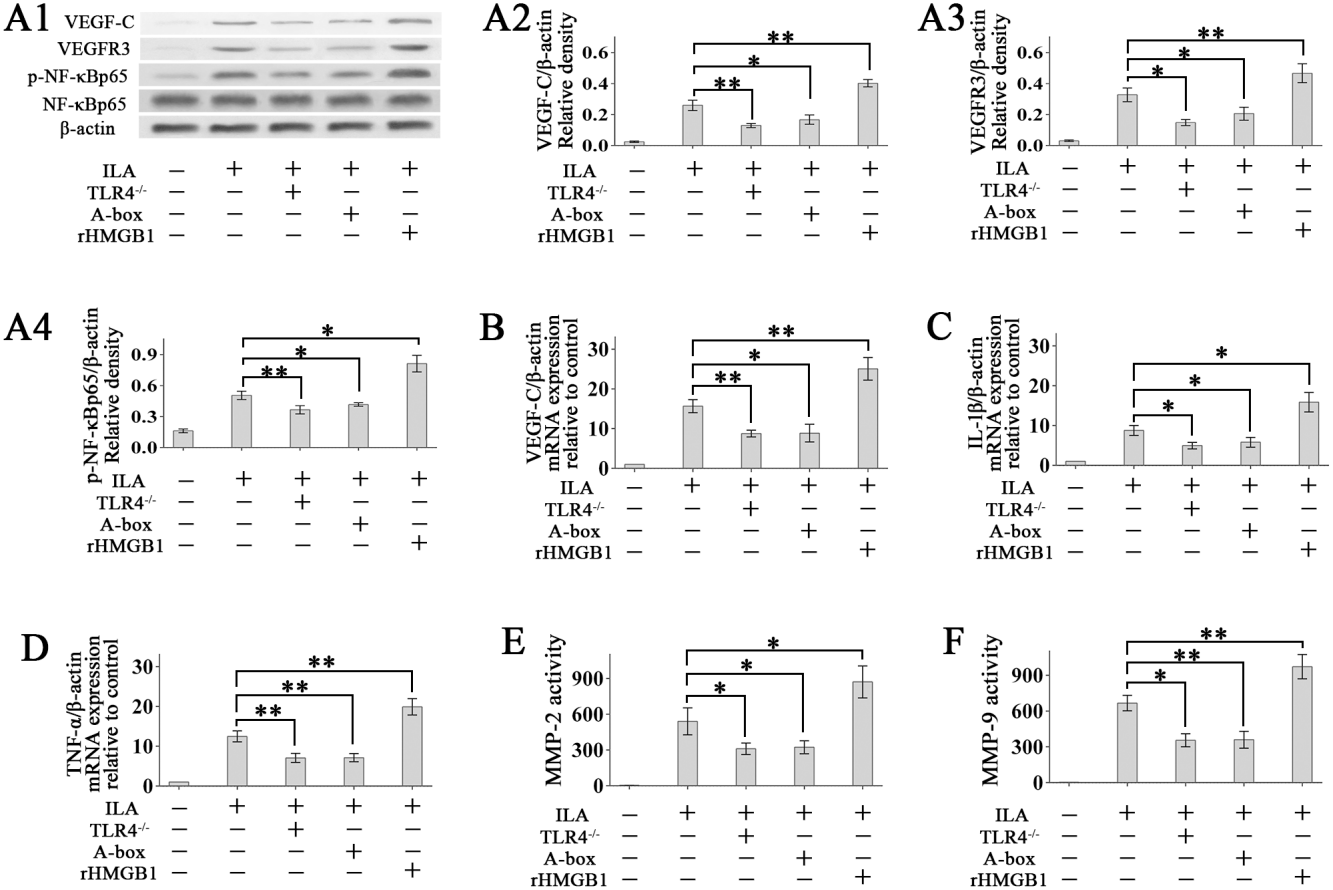
Multiple molecular mechanisms mediates HMGB1-TLR4 associated ILA. (A1-A4): The expressions of VEGF-C, VEGFR3 protein and the levels of phosphorylated and total NF-κBp65 in the ILA corneal lysates were detected using Western blotting. (B-D): The expressions of VEGF-C, IL-1β and TNF-α mRNA were detected using real-time PCR. (E and F): MMP-2 and MMP-9 activity in corneal lysates. *p < 0.05, **p < 0.01.

Firstly, the Knock-out of TLR4 or antagonizing HMGB1 by A-box down-regulated the VEGF-C/VEGFR3 expression and the phosphorylation of NF-κB p65, which were all up-regulated with rHMGB1-treatment (Fig 8A1–8A4 and 8B). Then, real-time PCR shows that knock-out of TLR4 or antagonizing HMGB1 inhibited the inflammatory factors IL-1β and TNF-α mRNA expression, and rHMGB1-treatmet increased IL-1β and TNF-α mRNA expression significantly (Fig 8C and 8D). Finally, knock-out of TLR4 or antagonizing HMGB1 by A-box inhibited MMP-2 and MMP-9 activity, and rHMGB1-treatmet increased MMP-2 and MMP-9 activity significantly (Fig 8E and 8F).

## Discussion

In the present study, we explored the role of TLR4 in HMGB1-associated ILA and mechanisms of HMGB1-TLR4 in modulating ILA. We found that HMGB1-associated ILA is primarily dependent on TLR4 but not on TLR2, and CD11b^+^ cells and multiple molecular mechanisms mediate HMGB1-TLR4 associated ILA. Furthermore, the inflammation-induced lymphangiogenesis can be effectively modulated by HMGB1-TLR4 signaling. This may provide us more opportunities to treat inflammation and lymphangiogenesis associated diseases.

Firstly, we find that the expression of HMGB1 and TLR4 increased notably in the ILA corneas compared with the normal corneas at both mRNA and protein levels, which shows that there may be a close relationship between HMGB1, TLR4 and ILA.

One of the most important findings in this study is the discovery that HMGB1-TLR4 modulated VEGF-C-induced LECs proliferation and tube formation in vitro. HMGB1 promoted VEGF-C-induced LECs proliferation in a dose-dependent manner when the concentration of HMGB1 was no more than 1000 ng/mL, which is similar to Yuanyuan Qiu’s study [13]. However, there are some differences between the two studies. In Yuanyuan Qiu’s study, there was no VEGF-C-induction when they gave HMGB1 treatment to LECs, and then the HMGB1 induced LECs proliferation with the maximal effect at a concentration of 2000 ng/mL [13].

The pre-incubation with HMGB1 increases the response of LECs to VEGF-C in the experiment. Kelly L. Hall, et al reported that LPS-induced activation of TLR4 increases the number of receptors (VEGFR3) for VEGF-C significantly, and LPS-treated RAW264.7 macrophages activate autocrine VEGF-C—VEGFR-3 loop [37]. This may explain why the response to VEGF-C is increased.

Next, the promoting effects of HMGB1 in LECs proliferation and tube formation were inhibited notably by knock-down of TLR4, which indicates that TLR4 mediates HMGB1-induced LECs proliferation and tube formation.

Accumulating studies indicate that HMGB1 and TLR4 strongly correlates with inflammation and lymphangiogenesis as we have already mentioned in the introduction. To explore the role of HMGB1-TLR4 in modulating inflammation-induced lymphangiogenesis systematically, we used a mouse ILA model, which shows that rHMGB1 treatment significantly promoted ILA, and the promoting effects was inhibited notably when HMGB1-TLR4 was blocked. Even without HMGB1 stimulation, ILA was also inhibited when HMGB1-TLR4 was blocked. This may be due to the fact that some HMGB1 was passively released from damaged cells when we made animal models, or was actively secreted by infiltrated immune cells in the following inflammatory processes [4–8]. These data suggest that TLR4 mediates HMGB1-associated ILA, and HMGB1-TLR4 signaling can effectively modulate inflammation-induced lymphangiogenesis.

HMGB1 signals have been reported through TLR2 and TLR4 [32], so we also analyzed the role of TLR2 in HMGB1-associated ILA. Our results demonstrate that the knock-out of TLR2 had no obvious influence on ILA in vivo, and the knockdown of TLR2 had no obvious influence on HMGB1-induced LECs proliferation and tube formation in vitro, regardless of the application of rHMGB1 or not. The above data suggest that HMGB1-associated ILA is primarily dependent on TLR4 but not on TLR2.

To explore the mechanisms of HMGB1-TLR4 in modulating ILA, we detected the levels of several key pro-lymphangiogenesis factors when we up-regulated or blocked HMGB1-TLR4 pathway.

Compelling evidence demonstrates that lymphatic endothelial cells in corneal ILA arise from CD11b^+^ cells [38]. CD11b is predominantly expressed in macrophages/monocytes and granulocytes [33–35]. It has been confirmed that macrophages/monocytes take crucial role in lymphangiogenesis [3, 38–40]. Macrophages are implicated as a source of lymphatic endothelial progenitors, and the pro-lymphangiogenic factors and proteolytic enzymes produced by actived macrophages promote ILA via multiple direct and indirect ways [37, 41–43]. In addition, recent researches demonstrate that neutrophils are also important contributors to lymphangiogenesis during inflammatory processes [44, 45]. In short, both macrophages/monocytes and granulocytes, the main CD11b^+^ cells, are important contributors to ILA. Therefore, we first detect CD11b^+^ cells in mechanisms.

Our present study shows that HMGB1-TLR4 played an important role in modulating the recruitment and activation of CD11b^+^ cells, which may be one of the most important cellular mechanisms in HMGB1-TLR4 associated ILA.

TLR4 in LECs is an essential molecule in the production of major chemokines for macrophages and in the subsequent recruitment of macrophages during LPS-induced lymphangiogenesis [46]. In addition, the induced macrophages by the LPS-TLR4 pathway in LECs contribute to robust lymphangiogenesis [46]. These researches are consistent with our conclusions.

It has been proved that the key pro-lymphangiogenesis molecules include VEGF-C/VEGFR3 signaling (the central regulator) [2, 47], inflammatory factors IL-1β and TNF-α [48], MMPs [49–51], and phosphorylated NF-κB p65 [28, 52], etc. Our in vivo experiments show that the above pro-lymphangiogenesis molecules were up-regulated in HMGB1-treatmet ILA corneas and were down-regulated in ILA corneas with Knock-out of TLR4 or antagonizing HMGB1 by A-box. These data suggest that multiple molecular mechanisms mediates HMGB1-TLR4 associated ILA.

In conclusion, to our knowledge, this study is the first to demonstrate systematically that HMGB1 promotes inflammation-induced lymphangiogenesis via TLR4-dependent signalling pathway and the ILA can be effectively modulated by HMGB1-TLR4 signaling. Consequently, administration of modulating inflammation-induced lymphangiogenesis through HMGB1-TLR4 pathway may provide more possibilities of treating inflammation and lymphangiogenesis associated diseases.

## Supporting Information

S1 Table Mouse primers used for Real-Time PCR experiments. (DOCX)
