# Supplementary material for: High Mobility Group Box-1 Promotes Inflammation-Induced Lymphangiogenesis via Toll-Like Receptor 4-Dependent Signalling Pathway
Source: PLoS One. 2016 Apr 21;11(4):e0154187. doi: 10.1371/journal.pone.0154187 (PMC4839690; doi:10.1371/journal.pone.0154187)
Supplement: S1 Table — (DOCX) [file pone.0154187.s001.docx]

**Supporting Information**

**S1 Table. Mouse primers used for Real-Time PCR experiments.**

| **Gene** | **Primer Sequence (**5’-3’) | **Hybrid. Temperature** | **Cycles** | **Product size (bp)** |
| --- | --- | --- | --- | --- |
| HMGB1 | Forward: GCATCCTGGCTTATCCATTGG | 61°C | 27 | 81 |
|  | Reverse: GGCTGCTTGTCATCTGCTG |  |  |  |
| TLR4 | Forward: AAATGCACTGAGCTTTAGTGGT | 60°C | 28 | 104 |
|  | Reverse:TGGCACTCATAATGATGGCAC |  |  |  |
| VEGF-C | Forward: TTTGCCAATCACACTTCCTGC | 57°C | 30 | 160 |
|  | Reverse: ACACTGTGGTAATGTTGCTGG |  |  |  |
| IL-1β | Forward: GCAACTGTTCCTGAACTCAACT | 57°C | 28 | 89 |
|  | Reverse: ATCTTTTGGGGTCCGTCAACT |  |  |  |
| TNF-α | Forward: CAGGCGGTGCCTATGTCTC | 58°C | 28 | 89 |
|  | Reverse: CGATCACCCCGAAGTTCAGTAG |  |  |  |
| β-actin | Forward: GGCTGTATTCCCCTCCATCG | 57°C | 21 | 154 |
|  | Reverse: CCAGTTGGTAACAATGCCATGT |  |  |  |
